# Understanding Experimental LCMV Infection of Mice: The Role of Mathematical Models

**DOI:** 10.1155/2015/739706

**Published:** 2015-10-21

**Authors:** Gennady Bocharov, Jordi Argilaguet, Andreas Meyerhans

**Affiliations:** ^1^Institute of Numerical Mathematics, Russian Academy of Sciences, Gubkina Street 8, Moscow 119333, Russia; ^2^Infection Biology Laboratory, Department of Experimental and Health Sciences (DCEXS), Universitat Pompeu Fabra, Doctor Aiguader 88, 08003 Barcelona, Spain; ^3^Institució Catalana de Recerca i Estudis Avançats (ICREA), Passeig Lluis Companys 23, 08010 Barcelona, Spain

## Abstract

Virus infections represent complex biological systems governed by multiple-level regulatory processes of virus replication and host immune responses. Understanding of the infection means an ability to predict the systems behaviour under various conditions. Such predictions can only rely upon quantitative mathematical models. The model formulations should be tightly linked to a fundamental step called “coordinatization” (Hermann Weyl), that is, the definition of observables, parameters, and structures that enable the link with a biological phenotype. In this review, we analyse the mathematical modelling approaches to LCMV infection in mice that resulted in quantification of some fundamental parameters of the CTL-mediated virus control including the rates of T cell turnover, infected target cell elimination, and precursor frequencies. We show how the modelling approaches can be implemented to address diverse aspects of immune system functioning under normal conditions and in response to LCMV and, importantly, make quantitative predictions of the outcomes of immune system perturbations. This may highlight the notion that data-driven applications of meaningful mathematical models in infection biology remain a challenge.

## 1. Introduction

**Figure 1:**
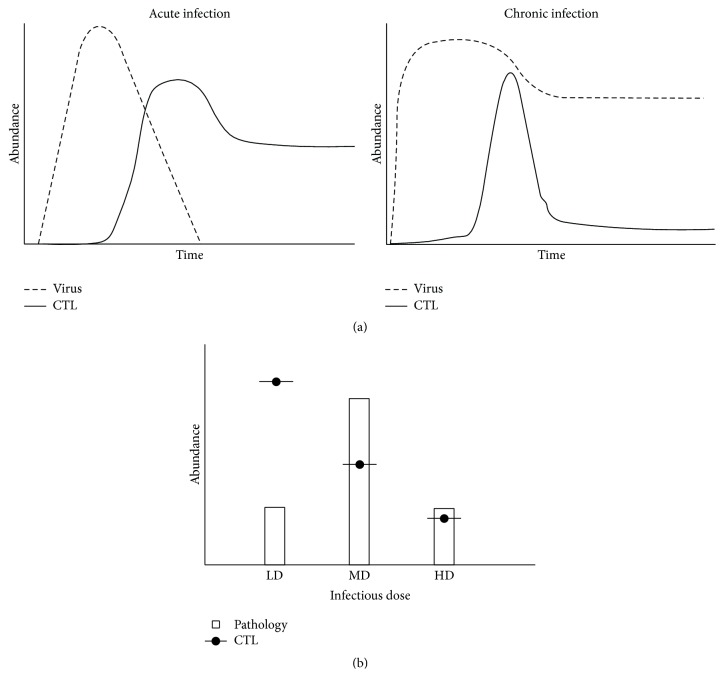
(a) Schematic view of acute and chronic LCMV infections including virus and cytotoxic T lymphocyte (CTL) dynamics. (b) Representation of the CTL-induced immunopathology dependence on the initial viral infectious dose at day 13 after infection (adapted from Cornberg et al. [62]). LD: low-dose infection; MD: medium-dose infection; HD: high-dose infection.

One of the best-studied model systems of viral infections is that of the lymphocytic choriomeningitis virus (LCMV) in mice (Figure 1) [1–3]. LCMV is an RNA virus of Arenaviridae that is noncytopathic in vivo. Thus, the virus itself does not cause direct damage to cells and tissues. This feature enables relating any damage that appears in the course of an infection to host responses against the virus. Another important feature of the LCMV model system is the existence of several well-characterized viral strains that differ in their replicative capacity, host range (cell tropism and mouse strain), and experimental routes of infection (intracranial versus intraperitoneal (i.p.) or intravenous (i.v.)) and thus show different infection outcomes. This enables directly linking easily measurable viral dynamic properties to pathogenic consequences and studying the fundamental issue of chronic infections.

With the use of the LCMV infection model system, a large number of conceptual discoveries in immunology have been made, of which we cite here just a few. First, back in 1974/75, Zinkernagel and Doherty demonstrated that cytotoxic T lymphocytes (CTLs) recognize foreign antigens only in the context of proteins of the major histocompatibility complex (MHC) [4, 5]. For this finding of “MHC restriction,” they were awarded the Nobel Prize in 1996. Second, with the help of knockout mice, the mechanism of CTL-mediated destruction of LCMV-infected target cells in vivo could be directly linked to perforin, a pore-forming protein contained in granules of this cell type [6, 7]. Third, fundamental properties of “memory” of the adaptive immune response have been studied. For example, a quantitative understanding on the number of epitope-specific precursor T cells, their expansion and contraction in the course of an acute LCMV strain Armstrong infection, and their maintenance was established [8–10] (for details, see below). Further studies defined the requirements for CTL memory to prevent the establishment of a persistent LCMV infection [11]. Fourth, NK cells of the innate immune response have been recognized as an important regulator of the helper T cell support for antiviral CTL [12]. Fifth, a critical role of organized secondary lymphoid organs in the induction of naive T and B cells and subsequent virus control was established [13]. Sixth, the concept of immunopathology, that is, the damage of tissues and organs due to the antiviral immune response rather than the infecting virus itself, was established. Mediators of immunopathology include CTL, macrophages, neutrophils, and interferons [14–16]. Seventh, based on the amino acid similarities between viral antigens and host proteins, the so-called molecular mimicry, viral infections can trigger autoimmunity and influence the course of subsequent infections with other viral pathogens [17–19]. Eighth, important observations towards an acute versus a persistent LCMV infection outcome were made [20–23]. Which infection fate is followed depends on the infecting viral dose and the infecting viral strain and thus can be easily directed experimentally. LCMV persistence is associated with CTL exhaustion, a reversible, nonfunctional state of CTL. As CTL exhaustion seems to be a physiological consequence of persistent antigen exposure and has been observed both in persistent human viral infections and in cancers, the LCMV system is highly attractive to understand infection fate regulation in general terms. As CTL exhaustion can be reversed by antibodies against PD1 or PD-L1 that block the negative signalling pathway, novel immunotherapeutic modalities arose which show exciting promises as antiviral and anticancer therapies [24–26]. Several clinical studies in this direction have been initiated and are ongoing.

The LCMV infection model system offers sufficient experimental data to develop mathematical models in a problem-oriented manner. Indeed, although only around 20 mathematical modelling studies have been published today addressing specific aspects of LCMV infection, they are more instructive than studies of other infections including HIV [27]. Indeed, the modelling studies of LCMV resulted in experimentally testable predictions concerning the mechanisms of the infection control, for example, (i) protective numbers of the initial CTL precursors to protect from a chronic LCMV infection outcome, (ii) minimal number of antigen presenting DCs in spleen for robust induction of CTL responses, and (iii) the effect of virus growth rate on the magnitude of the clonal expansion of CTLs, to name just the major of them. In this review, we summarize how the modelling approaches were tailored to address diverse aspects of immune system functioning under normal conditions and in response to LCMV and, importantly, make quantitative predictions of the outcomes of immune system perturbations.

## 2. Mathematical Models of LCMV Infection

Mathematical models developed for the analysis of experimental LCMV infection in mice enabled quantifying some fundamental parameters of the virus-host interaction including the numbers and turnover rates of immune cells and the growth rates of viruses. A maximum likelihood approach to nonlinear model parameter estimation utilizing precise and comprehensive data sets proved to be instrumental. The estimates of the fundamental parameters of T cell response to LCMV infection are summarized in Table 1.

### 2.1. Basic Numbers for Induction of the Clonal Expansion: Precursor CTLs and DCs

The initial frequency of virus antigen-specific T lymphocytes is a primary control parameter determining the speed and the magnitude of the antiviral immune response. Although the total number of lymphocytes in mice is high, that is, about 10^9^ [28], the fraction of the cells specific for a given viral antigen is very low being of the order of 10^−5^–10^−6^. As the division time of the lymphocytes is rather slow (~12 hours) compared to the replication rate of LCMV, the precise quantitation of the precursor T cell number is important in predicting the time needed for their clonal expansion above the threshold required to eliminate the virus from an infected animal. Early estimates of the number of LCMV-specific CTL precursor cells in spleen of naive C57BL/6 mice suggested that it is less than 1 in 10^5^ [29]. The first quantitative mathematical model of the LCMV infection provided the best-fit estimate of the number of LCMV Docile-specific precursor CTLs of about 27 cells per spleen [30]. A similar parameter estimation from experimental LCMV WE infections led to the value of about 110 naive virus-specific CTLs per spleen [31]. Taking into account the fact that the splenic population of lymphocytes is about 5% of the total lymphocyte number, the extrapolation from spleen to the whole mouse suggests that about 540 to 2200 precursor CTLs are specific for LCMV. This estimate is between the values obtained by later experimental examination of the precursor CTLs specific for the H-2D^b^-restricted GP33-41 (GP33) epitope of LCMV quantitated from in vivo competition assay [8] and those specific for an entire virus as quantitated via in vivo limiting-dilution assay [32]. The estimated numbers for naive C57BL/6 mice are from 100 to 200 GP33-specific CTLs to about 6,761 LCMV-specific CD8^+^ T cells, respectively. A finer quantitative dissection of CD4^+^ and CD8^+^ T cell responses to infection of C57BL/6 mice with LCMV Armstrong by considering the epitope-specific clones was done using the exponential growth and contraction model in [33]. The data-driven parameter estimation suggested the following population sizes for T cells starting the proliferation (i.e., for the lower bound on the precursor number) with their respective 95% confidence intervals (CI_95%_). For CD8^+^ T lymphocytes, the per-spleen estimates are 12 cells (3, 33) for GP33, 7 cells (1, 21) for NP396, 6 cells (0.4, 40) for GP118, 5 cells (1, 14) for GP276, 29 cells (9, 82) for NP205, and 165 cells (34, 519) for GP92 and in total ~224 cells per spleen. For CD4^+^ T lymphocytes, the estimates are 22 cells (19, 27) for GP61 and 56 cells (46, 74) for NP309 and in total 78 precursor cells. The above estimates were obtained by fitting the model to epitope-specific T cell data quantitated by intracellular cytokine staining. Similar parameter estimation from the data on Ag-specific CD8^+^ T cells in spleen measured by MHC tetramer staining resulted in the following numbers [34]: 8 cells (7, 11) for GP33, 5 cells (4, 6) for NP396, 2 cells (1.5, 3) for GP34, and 5 cells (4, 6) for GP276 and in total 20 cells.

The activation of T cells requires MHC-restricted antigen presentation by professional antigen presenting cells. The assessment of the threshold number of the dendritic cells for the induction of robust CD8^+^ T cell responses in secondary lymphoid organs was made using data-driven mathematical modelling. The study by Ludewig et al. [35] examined the impact of the dendritic cells (DCs) number on the induction of the CTL response. C57BL/6 mice were adoptively transferred by intravenous injection with 2 × 10^4^, 2 × 10^5^, and 2 × 10^6^ GP33-presenting DCs from transgenic mice ubiquitously expressing the LCMV glycoprotein peptide GP33 (H8-mice). The population dynamics of activated GP33-specific CTL (H2-D^b^/GP33-tetramer-binding, CD8^+^CD62L^−^) and quiescent “memory” CTL (CD62L^+^) in blood, spleen, and liver were followed. For the data analysis, a three-compartment delay differential equation model was formulated which considered the population dynamics of DCs and CTLs. The maximum likelihood approach to the model calibration was used to estimate the relevant parameter of the cell circulation and interaction. The model predicted that the threshold number of DCs in the spleen for induction of half maximal proliferation of CTLs is about 212 cells with the corresponding 95% uncertainty interval (75, 1200). A later independent study of the minimum number of DCs required to initiate a T cell response arrived at similar numbers [36]. The analysis combined the experimental assessment of the T cell and antigen-bearing DC encounters in popliteal lymph nodes (LN) with intravital 2-photon and confocal images and flow cytometry examination of the phosphorylation following the footpad injection of DCs and i.v. injection of CD4^+^ T cells and Dby peptides. The developed computational model of T cell DC encounter described the Brownian motion of moving T cell and static DCs in a spherical volume approximating the LN. The model allowed one to calculate the probability of a T cell to interact with antigen-bearing DC within 24 hours, which is 0.58 for 100 DCs and increases up to 0.99 for 10^3^ DCs, respectively.

### 2.2. T Cell Proliferation

Following the stimulation with LCMV antigens, the specific T lymphocytes enter the expansion phase and after reaching a peak of expansion the population starts to decline during the contraction phase. Although the general scales of the clonal expansion and contraction can be assessed directly from experiments with LCMV infection, for example, [21], the mathematical modelling in conjunction with the data on kinetics of the LCMV-specific T cell responses provided a fine kinetic characterization of the proliferation and death rates of the epitope-specific CD4^+^ and CD8^+^ T cells for both primary and chronic infection phases [33, 34, 37]. The data on LCMV-Armstrong i.p. infection of BALB/c mice were inverted into the estimates of the net doubling time of NP118 and GP283-specific CD8^+^ T cell and their half-life for the expansion and contraction phases of the primary immune response, respectively [37]. A piecewise linear system of ordinary differential equations was used to describe the population dynamics of naive, activated, and memory phenotype CD8^+^ T cells. A simplifying assumption was used saying that the viral load just switches the proliferation of the T cells between the full and zero modes in a time-dependent manner. The viral kinetics invariant estimates of the proliferation and death rates during the expansion and contraction phases, respectively, are presented as the doubling time and half-lives with the respective 95% uncertainty intervals. The best-fit doubling times are 5.7 (hours) (5.5, 6.2) for the NP118 epitope and 6.4 (hours) (5.5, 7.6) for the GP283 epitope. The best-fit half-lives are 32.6 (hours) (26, 39.6) for the NP118 epitope and 46.2 (hours) (19.8, 87.6) for the GP283 epitope.

A similar analysis has been used to assess differences in proliferation rates between CD4^+^ and CD8^+^ T cells during the clonal expansion phase [33]. The epitope-specific CD4^+^ and CD8^+^ T cells from spleens of C57BL/6 mice after intraperitoneal infection with 10^5^ pfu of LCMV Armstrong were used. Although it was pointed out that multiple mathematical formulations can be developed to describe the data, the information-theoretic criteria were not applied to rank the models. The best-fit estimates with the respective 95% confidence intervals obtained using a model similar to that of De Boer et al. [37] and extended to consider a biphasic contraction phase suggested the following doubling times for CD4^+^ and CD8^+^ T cells: for GP61-specific (immunodominant epitope) and NP309-specific (subdominant epitope) CD4^+^ T cells, the values were 11.3 (hours) (10.5, 12.1) and 15 (hours) (13.3, 17.3), respectively. The doubling times of CD8^+^ T cell were shorter with dominance ranking of GP33 > NP396 > GP118 > GP276 > NP205 > GP92 and were as follows: 8.8 (hours) (8, 9.6), 8.7 (hours) (7.8, 9.5), 8.9 (hours) (7.5, 10.4), 8.9 (hours) (8.1, 9.8), 10.9 (hours) (9.8, 12.1), and 14.7 (hours) (12.5, 16.7), respectively. The immunodominant epitopes exhibited faster proliferation rates.

### 2.3. Target Cell Elimination

It is not only the number of virus-specific CTLs that is important for the elimination of infection but also the efficacy of target cell elimination in vivo. The elimination rates of transferred cells expressing LCMV antigens into immune mice at the peak of an acute response and during the memory phase were first estimated using a simple exponential decay model in [38]. The best-fit estimates of the half-lives of the target cells at day 8 after infection were 1.4 hours and increased to 2.9 hours 30 days and finally to 8.9 hours 300 days after infection. The estimates of the elimination rates of the LCMV epitopes expressing cells by virus-specific CTLs in vivo were a subject of a number of follow-up studies in which more complex mathematical models were used.

Regoes et al. considered the migration of donor cells from blood to spleen [39]. The killing of the antigen-pulsed target cells by CTL in immune mice was assumed to follow a mass action law. The data used for the estimation of the target cell elimination rate were derived from experiments on i.p. infection of C57BL/6 mice with 2 × 10^5^ pfu LCMV Armstrong. The number of virus-specific CTLs at day 8 after infection was assumed to be 5 × 10^6^ per gram of spleen. The half-life of the PKH26-stained target cells due to the killing by NP396-epitope-specific CTL was estimated to be 0.17 (hours) with the 95% confidence interval (0.11, 0.33), whereas for the subdominant epitope the values were 0.33 (hours) (0.24, 0.46).

In the follow-up study [40] based on the same data, the data fitting procedure was refined to reduce the variability between animals (splenocyte numbers, magnitude of the CTL response to LCMV infection, and inocula size). This was done by (i) pairing the estimates of unpulsed and pulsed target cell frequencies in each animal and (ii) splitting the parameter estimation procedure into two stages, the estimation of the transfer rate of target cells from blood to spleen and the estimation of the target cell killing rate. This procedure led to about 3-fold increase of the best-fit estimate of the killing rate at the peak (day 8) of the acute infection.

The target cell elimination kinetics depend on many factors and processes including the migration of the cells into the spleen, the decay of the epitope from target cells, the number of antigen-specific CTLs, the functional status of CTL (i.e., effector state or exhaustion state), the load of LCMV peptides on target cells, and the parameterization of the target cell-CTL interaction. The last two issues have been systematically examined by Garcia et al. [41] following a model analysis oriented data generation approach. The in vivo killing assay was conducted in C57BL/6 mice acutely (200 pfu) or chronically (10^6^ pfu) infected with LCMV Docile. Six different peptide (GP33) loads spanning four orders of magnitude were used to pulse the adoptively transferred homozygous splenocytes. It was established that the ability of CTL to recognize and kill infected target cells depends on the number of peptide-MHC complexes presented on the cell surface. In addition, a saturation effect in target cell killing rates for high CTL numbers was suggested by the analysis of the quality of the data fitting. From the modelling perspective, a more accurate mathematical description of the target cell killing kinetics in relation to the peptide load (*λ*) and the abundance of CTL in the spleen (*C*) was proposed:

(1) $$\begin{array}{c} f\propto \frac{k_{max}\lambda }{\lambda_{0.5}+\lambda }\times \frac{C}{C_{0.5}+C}. \end{array}$$

The parameters characterizing the maximum killing rate, half-maximum peptide density, and half-maximum CTL frequency (*k*_max_, *λ*_0.5_, and *C*_0.5_) were estimated from the data. The minimal half-lives of infected cells (defined by the maximum killing rate in the respective groups) in face of their elimination by the epitope-specific CTLs were estimated to be 0.17 (hours), CI_95%_ = (0.11, 0.24), for acute infection, 0.32 (hours), CI_95%_ = (0.27, 0.37), for the memory phase, and, surprisingly, 0.38 (hours), CI_95%_ = (0.31, 0.44), for the chronic infection phase. The value of *λ*_0.5_ characterizing the sensitivity of CTL to the peptide frequency of the presented epitopes on the target cells was estimated to be the highest in acute infection and similar for the chronic infection and the memory infection phase. Furthermore, the limits of validity of the mass action law in the description of the target cell elimination, as generally used in data analysis, were examined. The values of the CTL abundance in spleen were estimated for acute infection, memory infection phase, and chronic infection to be 0.042, 0.031, and 0.006, respectively. Earlier computational studies based on a cellular automata model predicted that, above CTL frequencies of 0.03, saturation effects of target cell elimination have to be taken into account [42]. The estimated values of the threshold density of CTL *C*_0.5_ at which the elimination rate is half-maximum are 0.013 with CI_95%_ = (0.004, 0.023) for acute and chronic infection but increase 4-fold to 0.051, CI_95%_ = (0.007, 0.088), for the memory phase of infection. Overall, the study led to a number of novel insights into the mechanics of target cell elimination: (i) there is no evidence of an increased recognition sensitivity of memory CTL compared to acute or chronic CTLs; (ii) the killing ability of CTL in chronic LCMV infection is at least as strong as during acute infection.

## 3. Acute and Chronic LCMV Infection

There are relatively few mathematical models of LCMV infection in which the population dynamics of viruses and immune responses as shown in Figure 1 were formulated [30, 43–45]. The validity of the law of mass action in the description of target cell elimination by CD8^+^ T cells was shown [43]. The mathematical model formulated with ODE described the growth and elimination of the virus population by CTLs. The dynamics of the adoptively transferred LCMV peptide-loaded target cells were described analytically following the model by Ganusov and De Boer [46]. An exponential growth model was used to describe the expansion of CTLs. The combined equations allowed the authors to estimate the critical number of CTLs at which the virus growth can be prevented from the start of infection. For LCMV Armstrong with the exponential growth rate assumed to be 5 per day, the protective number of memory CTLs was around 1.3 × 10^5^ cells.

One of the first quantitative models of LCMV infection was developed by Bocharov [30] to describe the population dynamics of virus, precursor CTL, and effector CTL using delay differential equations. The data in low-, intermediate-, and high-dose infection of C57BL/6 mice with LCMV Docile [21] were used for model calibration. The model was used to predict the effect of the variation in the number of precursor CTLs on the outcome of LCMV infection. The model-generated predictions were tested experimentally by Ehl et al. [11] and the following conclusions have been made: (i) a minimal threshold number of about 25–50 naive LCMV-specific CTL precursors (CTLp) are necessary for control of infections in the range of 1–10^4^ pfu; (ii) with 10-fold higher doses, a 100-fold increase in CTLp is required to restore virus control; (iii) in high-dose infection (above 10^6^ pfu), elevations in CTLp were found to be detrimental as they changed the outcome of infection from harmless virus persistence to lethal immunopathology. Overall, above a critical threshold, the time when effector function is reached by CTLs is more important than the initial number of virus-specific CTL precursors.

The mathematical model developed by Bocharov [30] was further used to predict the impact of the virus replication kinetics on the magnitude of the CTL response in acute LCMV infection. The experimental analysis of the clonal expansion of CTLs in C57BL/6 mice to LCMV strains (Armstrong, WE-Armstrong, WE, Traub, and Docile) differing in their replication rate [47] confirmed that there is a bell-shaped relationship between the LCMV growth rate and the peak CTL response. It was shown that both slow and fast replicating LCMV strains produce weaker CTL responses. A mechanism of virus persistence by sneaking through immune surveillance due to slow replication kinetics was hypothesized and its relevance for HBV and HCV infections was shown. The “underwhelming” infection mechanism (supplementing the “overwhelming” infection [21]) fits the concept of the sensitivity of immune responses to perturbations [48].

Infection of mice with certain strains of LCMV can result in the development of lifelong virus persistence. The role of various host and viral parameters in the development of chronic LCMV infection has been examined experimentally. One of the fundamental features of the establishment of LCMV persistence was associated with the exhaustion of antiviral cytotoxic effector T cells after their early and complete induction [21]. The phenomenon of exhaustion was defined as complete disappearance of CTL activity and the clonal deletion of virus-specific CTLs. The exhaustion of antiviral CTL responses was a stepwise process observed in an overwhelming infection with LCMV Docile or LCMV Clone 13. Following the initial activation, LCMV-specific T cells become anergic for 3 to 5 days and then disappear because of activation-induced cell death (apoptosis). (Of note, the observed lack of T cell functionality was in time of the described experiments termed “anergy”; however, this functional state of T cells was subsequently studied in more detail and shown to be a nonresponsive state after continuous antigen exposure that is now termed “exhausted”; for a detailed discussion, see Wherry and Kurachi [49].) The phenomenology of conventional and exhaustive CTL responses was quantitatively described in the mathematical model by Bocharov [30]. The single characteristic that appeared to be sufficient to control conventional versus exhaustive responses of CTLs was the cumulative viral load since the beginning of the infection *W*(*t*) = ∫_0_^*t*^ *V*(*s*)*ds*. The increase of *W* above a certain threshold value in conjunction with the high viral load in the host for about 5 days results in the shift of the infection phenotype from an “acute with recovery” to a chronic infection. The model allowed estimating the fraction of virus population homing to spleen (*V*_spleen_) as a saturating function of the inoculum size (IS):

(2) $$\begin{array}{c} V_{Spleen}=\frac{0.37\times \text{IS}}{\left(1+\text{IS}/\left(0.84\times 10^{5}\right)\right)}. \end{array}$$

The model was used to generate biologically relevant predictions amenable to experimental testing: (i) the impact of the precursor CTL number on the dynamics of LCMV infection and (ii) the effect of the virus growth rate on CTL expansion. A bifurcation analysis of the model was used to specify the parametric conditions of low level LCMV persistence after an acute infection [50]. In addition, extensions of the model were used to theoretically examine the efficacy of protection and immunopathology by effector memory versus naive CTLs against intravenous or peripheral infections [31], the role of antigen-specific versus bystander stimulation for persistence, and the structure of CTL memory in LCMV infection [51].

Models of LCMV infections of mice are an example of rival approaches to the description of the exhaustion phenomenon. While the Bocharov models assumed CTL anergy and apoptosis, two other models used nonoverlapping assumptions: (1) the virus infecting APCs and CD4^+^ T cells that are later killed by CTLs, thus negatively affecting the clonal expansion loop [45], or (2) the direct competition between the innate immunity and CTLs that is mediated by direct elimination of CTLs by innate immunity and indirect inhibition of CTLs by elimination of the antigen [44]. It is interesting to note that very recent studies demonstrated a direct and an indirect contribution of innate NK cells to T cell exhaustion during primary and chronic infection phases [12, 52–54]. A model refinement based on these new findings seems worthwhile.

The LCMV polymerase is error prone. Escape mutations in the viral envelope of LCMV have been considered as another factor in the establishment of chronic infections when acute CTL responses fail to eliminate the virus. Initially, the physical deletion of CTLs was attributed to the exhaustion phenomenon [21]. Recent studies have suggested that the exhaustion phenotype results from a gradual process and is associated with long-term persistence of CTLs with a reduced functionality, that is, the presence of mono- or bifunctional CTL populations [55]. The overall approach was model driven and focussed on the analysis of the protective efficacy of individual CTL specificities in chronic infection of C57BL/6 mice coinfected with wild type LCMV Cl13 and mutant viruses (CTL epitope mutants: GP33, NP396, and GP276). The mathematical model for the population dynamics of the mutant and wild type (WT) viruses was used to quantify the epitope-specific CTL selection pressure in chronically infected mice. The kinetics of the selection pressure exhibited extensive diversity between individual mice. However, the CTL selection pressure was not lost during the chronic phase of the infection. The early onset of the CTL pressure on the GP276 and GP33 epitopes was documented with the action of GP276 during the first 50 days of persistent infection and the biphasic model of GP33-specific selection pressure. The NP396-specific CTLs were documented to become relevant for selection in a later stage between days 30 and 80 after infection. Interestingly, lack of correlation between the GP276-specific CD8 T cell frequencies in peripheral blood and epitope-specific CTL pressure was observed. Thus, the conventional approach to study the immune correlates of CTL efficacy in terms of avidity, proliferative capacity, perforin expression, resistance to immune regulation, and so forth by monitoring the cells in peripheral blood seems to be not informative enough. Because of the skewing of virus-specific CTLs to LCMV-infected tissues, it is necessary to assess the cell functionality in a tissue-related manner. To overcome the above limitations, novel methodological approaches based upon the analysis of gene expression profiles in conjunction with markers of CTL efficacy, that is, global transcriptome examinations of infected organs, are needed. These issues obviously represent a challenge to mathematicians in terms of the computational tools that are needed for big-data- and multiscale modelling of LCMV-host interaction dynamics quantitated by using modern high-throughput experimental technologies.

## 4. Model Ranking and Selection

The interaction between a virus and the immune system can be described by multiple mechanisms using various types of modeling formalism (differential equations, cellular automata, and lumped versus spatial considerations). Furthermore, immunological and mathematical knowledge enter models a priori in the form of simplifying assumptions. However, a major limitation is that these assumptions about biological processes are often incompletely understood and the consequences of the necessary simplifications are therefore difficult to predict. Modeling the LCMV infection of mice is an example of the above dilemma.

Three essentially differing mathematical models of the virus-immune response dynamics were developed to explain the phenomenon of CTL exhaustion, reflecting the fact that translation of an immunological phenomenon into a mathematical structure is a nonunique procedure [30, 44, 45]. A computationally intense analysis of various model formulations (30 variants) for the regulation of immune responses in LCMV infection was presented by Rouzine et al. [56]. The ranking was based upon the mean square deviation and the analysis of the Akaike criterion for model and experimental data on LCMV infection in BALB/c and 129/ScEv mice with 4 different strains (Armstrong, Docile, Clone 13, and Aggressive). The best-fit model of the CTL regulation is characterized by a linear control function of the activation process by APCs.

In the process of model development and calibration, some principles have to be considered [57]. A basic requirement is a computational methodology for discriminating between rival models that are constructed from observed data. If there are a number of candidate models, the task is not simply to identify the one with the smallest least squares deviation function but to consider the principle of parsimony for the maximum use of information implicit in the data. If one has confidence in the forms of nested models, one criterion by which to rank them may be the size of the objective function. Model discrimination for nested models is based upon standard hypothesis tests such as the *F*-test. Following an information-theoretic approach to model building one quantitates the information lost when the model is used to approximate the reality or “full truth.” The ranking methodology is that associated with minimum information loss. The latter expression is taken here in terms of the Kullback-Leibler information-theoretic measure of the “distance between” two probabilistic models. It provides a basis for deriving “information-theoretic” criteria such as the Akaike, Schwarz, and Takeuchi indices [58]. The minimal value of the Akaike index suggests that the preferred model ensures a balance between overabundance of parameters (overfitting the data) and sparsity of parameters (underfitting the data). The minimum description length (MDL) provides a selection method that is sensitive to a model's functional form and favors the model that permits the greatest compression of data in its description [59]. Though well established, only three modelling studies of the LCMV infection utilized the information-theoretic approach to model ranking [40, 41, 56, 60].

The mathematical models for the virus-CTL interaction in acute LCMV infections (see Figure 1) can be defined within a set of two- or three-dimensional ordinary differential equations (ODEs) or delay differential equations (DDEs) representing the dynamics of the virus and that of virus-specific CTL (activated and memory cells) populations. It is remarkable that the best-approximating model according to the Akaike criterion for the typical data set of LCMV-CTL population dynamics in primary infections appears to be the one which was introduced elsewhere in an ad hoc manner [33, 37]. Specifically, the parsimonious form of the proliferation term implies that the CTL response to a low-dose LCMV infection is a process regulated by the virus load in an “on” (full activation) and “off” (no activation at all) way.

## 5. Conclusions

In conclusion, the mathematical modelling approaches to LCMV infection in mice provided rate estimates for T cell turnover, infected target cell elimination, and precursor frequencies but also shed new light on quantitative relationships or “the numbers game” between the virus and the host [28]. This represents the level of virus and cell population dynamics. However, in the immune system, complexity exists on additional levels including the single cell level with tunable responsiveness and the level of the complete host with its anatomical context [48]. With the growing body of high-throughput data from various perturbation experiments at the molecular, the cellular, and the tissue level, the field will be dependent on the ability to develop innovative computational methodologies for data assimilation, analysis, and predictions. A necessary prerequisite is a tight collaboration and mutual understanding between mathematicians and immunologists. There are richness of opportunities and myriads of challenges [61].

## Figures and Tables

**Table 1:** Fundamental parameters of T cell response to LCMV infection and the relevant LCMV epitopes.

Parameter (mouse strains)	Estimated parameter: value, range (CI_95%_), union of ranges	References
Number of single LCMV-epitope-specific precursor CD8^+^ T cells (C57BL/6 mice)	100–200 (per mouse): GP33 5–165 (per spleen): GP33, GP92, GP118, GP276, NP205, and NP396 2–8 (per spleen): GP33, GP34, GP276, and NP396	[8, 32–34]

Number of single LCMV-epitope-specific precursor CD4^+^ T cells (C57BL/6 mice)	22–56 (per spleen): GP61, NP309	[33]

Total number of the precursor CD8^+^ T cells specific for an entire virus (C57BL/6 mice)	27–110 (per spleen): WE and Docile 6,761 (per mouse): GP33 + GP118 + GP276 + NP396 + NP205 224 (per spleen): GP33 + GP118 + GP276 + NP396 + NP205 20 (per spleen): GP33 + GP34 + GP276 + NP396	[30–34]

Total number of the precursor CD4^+^ T cells specific for an entire virus (C57BL/6 mice)	78 (per spleen): GP61 + NP309	[33]

Number of dendritic cells required for induction of CD8 T cell clonal expansion (C57BL/6 mice)	212, CI_95%_ = (75, 1200) (per spleen): H8-mice DCs	[35]

Doubling time of LCMV-epitope-specific CD8^+^ T cells during clonal expansion phase (C57BL/6 mice, BALB/c mice)	7.5–16.7 (hours): GP33, GP92, GP118, GP276, NP205, and NP396 5.5–7.6 (hours): GP238, NP118	[33, 37]

Doubling time of LCMV-epitope-specific CD4^+^ T cells during clonal expansion phase (C57BL/6 mice)	10.5–17.3 (hours): GP61, NP309	[33]

Half-lives of epitope-specific CD8^+^ T cells during contraction phase (BALB/c mice)	19.6–87.6 (hours): GP238, NP118	[37]

Half-lives of infected target cells killed by epitope-specific CD8^+^ T cells (C57BL/6 mice)	1.4 (hours) at day 8, 2.9 (hours) at day 30, and 8.9 (hours) at day 300: H8-spleen cells 0.11–0.46 (hours) at day 8 with effector frequency 0.05 per spleen: GP276, NP396 0.048–0.16 (hours) at day 8: GP276, NP396 0.11–0.24 (hours) for acute infection (day 8), 0.27–0.37 (hours) for memory phase (day 42), and 0.31–0.44 (hours) for chronic infection (day 42): GP33	[38–41]

Threshold frequency of CTLs in spleen at which the infected cells elimination rate is half-maximum (C57BL/6 mice, infection with LCMV Docile)	0.004–0.023 for acute (day 8) and chronic (day 42) infection, 0.007–0.088 for memory phase of infection (day 42): GP33	[41]

Protective number of memory CTLs against infection (C57BL/6 mice, infection with LCMV Armstrong)	1.3 × 10^5^ cells per spleen for GP276, NP396	[43]

Protective number of naive precursor CTLs against chronic infection (C57BL/6 mice)	10^5^ cells per spleen for infection with 10^5^ pfu LCMV Docile (cells from TCR318 mice)	[11]

Dependence of CTL clonal expansion on virus growth rate (C57BL/6 mice)	Bell-shaped; both slow and fast replicating virus strains can induce weak CD8^+^ T cell clonal expansion (GP33)	[47]
